# Metacognitive Monitoring in Reading Comprehension: Examining the Role of Cognitive Flexibility, Vocabulary, and Fluency in Young Readers

**DOI:** 10.3390/jintelligence14030042

**Published:** 2026-03-05

**Authors:** Vered Markovich, Shoshi Dorfberger, Vered Halamish, Tami Katzir, Dana Tal, Rotem Yinon

**Affiliations:** 1 Edmond J. Safra Brain Research Center for the Study of Learning Disabilities, University of Haifa, Haifa 3103301, Israel; katzirta@gmail.com (T.K.); codana7@gmail.com (D.T.); rotemwr82@gmail.com (R.Y.); 2 Department of Special Education, Gordon Academic College of Education, Haifa 3570503, Israel; shoshid@gordon.ac.il; 3 Faculty of Education, Bar-Ilan University, Ramat Gan 5290002, Israel; vered.halamish@biu.ac.il

**Keywords:** metacognitive monitoring, calibration accuracy, reading comprehension, vocabulary knowledge, reading fluency, cognitive flexibility

## Abstract

This study examined associations between vocabulary knowledge, reading fluency, cognitive flexibility, and metacognitive monitoring accuracy in reading comprehension among fifth-grade students. Participants (N = 104) completed measures of cognitive–linguistic abilities and reading comprehension, with global metacomprehension judgments after reading and item-level confidence ratings. Metacognitive monitoring accuracy was assessed using calibration of global metacomprehension judgments and item-level confidence ratings. Calibration bias (confidence minus performance) indexed miscalibration direction, and its absolute value indexed calibration accuracy. Resolution reflected discrimination between correct and incorrect item-level responses. Structural equation modeling (SEM) was used exploratorily to examine theoretically motivated direct and indirect pathways via reading comprehension. Vocabulary knowledge showed the strongest associations with calibration accuracy and resolution, fully mediated by comprehension. Reading fluency showed a dual pattern: it contributed positively to resolution through comprehension, while also showing direct associations with lower calibration accuracy, indicating greater miscalibration and overconfident judgment tendencies among more fluent readers. Cognitive flexibility was not significantly related to any monitoring index. By jointly examining distinct indices of monitoring accuracy and separating comprehension-mediated from direct pathways, the study clarifies how cognitive–linguistic abilities may support or bias metacognitive monitoring in developing readers. Linguistic abilities, particularly vocabulary and fluency were central to students’ comprehension monitoring accuracy.

## 1. Introduction

Metacognitive monitoring in reading refers to the ability to assess and track one’s understanding during text processing, and it is considered a central aspect of self-regulated learning (Golke et al., 2022). In developing readers, effective monitoring enables children to evaluate their understanding of texts, detect comprehension failures, and adaptively adjust their reading strategies (Mirandola et al., 2018). Despite its importance, the cognitive mechanisms underlying individual differences in metacognitive monitoring accuracy while reading remain insufficiently understood. In particular it is unclear how different cognitive–linguistic abilities may support or bias monitoring judgments through distinct pathways. To address this gap, the present study extends the literature by examining both global and item-level calibration, resolution, and mediation via reading comprehension. Given the transparent yet morphologically rich nature of Hebrew orthography, the present study also contributes to understanding metacognitive monitoring in reading within a linguistically distinctive context.

Metacognitive monitoring accuracy is measured through two complementary indices. Calibration reflects the absolute correspondence between confidence judgments and actual performance, whereas resolution captures the ability to distinguish between correct and incorrect responses based on confidence levels (Dunlosky & Thiede, 2013; Nelson & Narens, 1990). Both indices reliably predict academic achievement (Stankov et al., 2015; Yang et al., 2022). Calibration can be decomposed into a directional bias index, capturing overconfidence versus underconfidence (confidence minus performance). It can also be used as an accuracy index reflecting the absolute magnitude of miscalibration, defined as the absolute discrepancy between confidence and performance (Schraw, 2009; Temelman-Yogev et al., 2020).

Calibration judgments may be obtained at different stages of reading, either globally before testing or at the item level during performance. This distinction highlights substantial individual differences in monitoring accuracy, with some students consistently overestimating or underestimating their performance, whereas others demonstrate more accurate self-evaluations. These patterns reflect meaningful developmental variation in metacognitive monitoring during middle childhood (Mirandola et al., 2018; Roebers, 2017). Crucially, the distinction between global metacomprehension judgments and item-level confidence judgments is conceptual and temporal rather than merely a matter of granularity. Global judgments made before testing reflect readers’ overall sense of understanding, whereas item-level ratings collected during the test are tied to specific response decisions and may engage different monitoring processes and cues (Schraw, 2009). Building on foundational metacognitive models, the present study adopts Koriat’s (1997) cue-utilization framework to conceptualize metacognitive monitoring in reading comprehension. Accordingly, monitoring relies on inferential processes based on the availability and informative value of cues. Monitoring accuracy thus depends on the individual’s ability to detect and utilize cues that reliably indicate task performance. At the same time, it requires avoiding overreliance on less informative cues. In the context of reading comprehension, relevant cues may include textual coherence, vocabulary familiarity or processing fluency (the ease and speed of reading). Although fluency is often a helpful cue, it may become misleading in cases where processing ease is dissociated from actual comprehension. This raises the possibility that fluency may operate as a dual-purpose cue: it may support comprehension when processing reflects meaningful integration, but it may also lead to inflated confidence when readers rely on it heuristically as a proxy for understanding.

In this study, cue use is discussed conceptually rather than assessed directly. Against this background, vocabulary knowledge and reading fluency are expected to relate to monitoring accuracy through different pathways. Specifically, vocabulary provides access to semantic cues that support accurate monitoring, whereas fluency may be associated with facilitation of comprehension or with biased monitoring judgments, depending on whether it reflects meaningful processing or superficial ease. Cognitive flexibility may still contribute by supporting adaptive cue evaluation and error detection, but its role may be attenuated when domain-specific linguistic resources are strong. By jointly examining calibration and resolution, this framework addresses a critical gap in understanding mechanisms underlying metacognitive monitoring in developing readers (Mirandola et al., 2018; Roebers, 2017; Undorf et al., 2018; Undorf & Bröder, 2020). The present study therefore examines how vocabulary knowledge and reading fluency differentially contributes to two forms of calibration (global and item-level) and to resolution, and whether these associations are mediated by reading comprehension. This approach is grounded in evidence that vocabulary and fluency support comprehension through distinct mechanisms (Cain et al., 2004; Wright & Cervetti, 2017), and in findings that comprehension itself underlies the development of accurate monitoring (Roebers, 2017). The goal is to clarify how key cognitive–linguistic skills support or bias fifth-grade students’ monitoring accuracy during a critical developmental period in which students are expected to comprehend increasingly complex texts and begin to engage in independent self-monitoring (Roebers, 2017). At the same time, large individual differences in monitoring accuracy are well-documented at this age, making it an ideal developmental window for examining how cues are used and misused.

### 1.1. Cognitive–Linguistic Resources Supporting Metacognitive Monitoring

This section reviews key linguistic and cognitive factors that may shape metacognitive monitoring during reading. Primary focus is placed on vocabulary knowledge and reading fluency, with secondary consideration of cognitive flexibility and working memory. These constructs were selected for their theoretical relevance to cue-based monitoring and their documented links to reading comprehension (Cain et al., 2004; Kim, 2020; Morris & Lonigan, 2022; Peng et al., 2018; Perfetti, 2007; Roebers, 2017). Each is discussed in relation to its potential role in supporting students’ ability to evaluate their understanding accurately.

**Vocabulary knowledge** may function as a foundational resource for metacognitive monitoring through several interconnected theoretical mechanisms. Vocabulary represents a critical component in establishing lexical standards for monitoring reading comprehension, as gaps in lexical knowledge may lead to failures in detecting inconsistencies in text (Cain et al., 2004). Longitudinal evidence further shows that vocabulary knowledge predicts children’s later ability to distinguish correct from incorrect responses in math and science tasks, pointing to domain-general and developmental links between lexical knowledge and monitoring processes (Buehler et al., 2025). The theoretical link between vocabulary knowledge and metacognitive monitoring is strengthened by the “lexical quality hypothesis” (Perfetti, 2007), which posits that highly developed lexical representations free up cognitive resources that can be allocated to higher-level processes as metacognitive monitoring. When lexical access is rapid and automatic, readers can devote greater cognitive capacity to evaluating comprehension accuracy and detecting semantic inconsistencies.

**Reading fluency** can exert both supportive and biasing effects on metacognitive monitoring. In its supportive role, fluency facilitates efficient word recognition and reduces cognitive load. This allows readers to allocate more resources to meaning construction and inferential processing. Evidence suggests that improvements in fluency can enhance comprehension processes without necessarily deepening semantic interpretation. For example, Juhkam et al. (2023) found that although both fluency and metacognitive knowledge improved through reciprocal teaching, only gains in metacognitive knowledge predicted comprehension. These findings imply that fluency supports comprehension primarily by increasing processing efficiency. Similarly, a meta-analysis by Burns (2024) demonstrated that fluency interventions, especially when tailored to students’ accuracy levels, enhanced comprehension in early readers (Grades 1–3). Thus, fluency appears to support comprehension primarily through processing efficiency and reduced effort, rather than through deeper semantic integration. When fluency effectively supports comprehension, it may also strengthen resolution, as deeper comprehension provides more reliable item-level cues that help confidence align with accuracy. In contrast, effects on calibration are less certain given that calibration reflects the alignment between average confidence and performance.

On the other hand, fluency may be associated with systematic biases in metacognitive judgments. Consistent with the cue-utilization framework outlined above (Koriat, 1997), fluency may also introduce systematic biases in metacognitive judgments when interpreted heuristically. This may result in an “illusion of knowing”, particularly when fluency and comprehension are misaligned. For example, high fluency with shallow understanding can inflate confidence (overestimation), whereas low fluency with adequate understanding can depress confidence (underestimation). In this context, confidence judgments may reflect the influence of such heuristic cues more than fluency itself. Thus, even though fluency typically predicts comprehension, its heuristic interpretation may be associated with lower monitoring accuracy. Within this framework, fluency-related effects are examined as correlational associations, without assuming direct evidence of heuristic cue use. This effect is most evident in distorted calibration and, in some cases, weakened resolution when comprehension is not aligned with fluency. Accordingly, fluency can enhance resolution indirectly through its contribution to comprehension, while simultaneously posing risks for miscalibration when relied upon heuristically. Indeed, research has shown that even among readers with comparable fluency levels, calibration accuracy can vary substantially, underscoring the importance of examining both trait-based and task-related factors that influence monitoring accuracy (Burns, 2024; Juhkam et al., 2023).

Taken together, reading fluency may function as a dual cue: it can serve as a cognitive resource that supports resolution through its contribution to comprehension, but it may also act as a misleading heuristic that inflates confidence when used as a surface-level indicator of understanding. The present study therefore emphasizes the need to examine both the indirect (resource-based) and direct (non-mediated) pathways linking fluency to monitoring accuracy.

**Cognitive flexibility** represents the capacity to shift mental sets and adapt to changing task demands (Fiske & Holmboe, 2019; Friedman & Robbins, 2022). Cognitive flexibility is widely recognized as a critical aspect of general intellectual functioning, influencing an individual’s ability to navigate complex problem-solving, learn from experience, and adapt to novel situations across various cognitive domains (Birney & Beckmann, 2022; Schmitz & Krämer, 2023). Theoretically, cognitive flexibility may enhance metacognitive monitoring by supporting error detection, strategic adjustment, and inhibitory control over premature confidence judgments. Within a cue-utilization framework, cognitive flexibility may assist students in evaluating the diagnostic value of available cues. It may allow them to down-weight superficial cues such as ease of processing and up-weight semantic and contextual cues that more reliably indicate comprehension. In addition, it may facilitate accurate cue evaluation by enabling attentional shifts, suppression of misleading cues such as superficial fluency, and updating of mental representations based on more reliable semantic or syntactic information. Recent evidence links cognitive flexibility to early literacy outcomes (Tal & Shaul, 2024), yet its contribution to reading comprehension monitoring remains unclear. Research suggests that reading-specific flexibility (coordination among phonological, orthographic, and semantic processes) predicts reading outcomes more consistently than domain-general flexibility (Cartwright et al., 2019). Thus, in the present study, cognitive flexibility is examined as a secondary exploratory factor. It is expected to contribute modestly to monitoring accuracy once core linguistic resources such as vocabulary knowledge and reading fluency are accounted for, rather than functioning as a regulatory mechanism tested through interaction effects. This approach aligns with reading comprehension models that emphasize the central role of linguistic resources.

Taken together, these cognitive–linguistic resources do not operate in isolation but are embedded within broader theoretical models of reading comprehension that specify how linguistic knowledge and executive regulation jointly support comprehension and monitoring. Reading comprehension is increasingly conceptualized as the coordinated outcome of linguistic skills and higher-order cognitive regulation. According to the **Simple View of Reading**, comprehension reflects the joint contribution of word reading efficiency and language comprehension, with language-based skills such as vocabulary becoming more central as decoding is automatized (Gough & Tunmer, 1986; Hoover & Gough, 1990). Extending this account, the **Not-So-Simple View of Reading** incorporates domain-general cognitive processes, including working memory and executive coordination, as integral contributors to comprehension (Berninger & Winn, 2006). More recently, the Direct and Indirect Effects Model of Reading (DIER) specifies that linguistic skills exert direct effects on comprehension, whereas executive functions influence comprehension indirectly by regulating the deployment of these skills under demands for integration, inference, and monitoring (Kim, 2020). Prior research links working memory to reading comprehension. However, integrative developmental models suggest that its direct contribution decreases beyond early reading stages and becomes largely indirect once core linguistic skills are considered (Kim, 2020; Morris & Lonigan, 2022; Peng et al., 2018). Therefore, working memory was included in the current study as a secondary, exploratory executive-function control rather than a focal predictor. It indexed individual differences in maintaining and manipulating verbal information during comprehension and monitoring.

### 1.2. Individual Differences and Development

Evidence from the Dunning–Kruger effect (Dunning et al., 2003; Mazor & Fleming, 2021) suggest that lower-performing individuals may lack the metacognitive insight needed to assess their performance reliably. In the context of reading, this implies that individual differences in vocabulary knowledge, reading fluency, and cognitive flexibility may affect monitoring accuracy not only by supporting comprehension but also by shaping students’ attention to diagnostic cues and their strategic use of cognitive resources during self-evaluation (Koriat, 1997; Perfetti, 2007; Roebers, 2017). Developmental research has shown age-related improvements in children’s metacognitive monitoring abilities and persistent individual differences across elementary school years (Koriat & Ackerman, 2010; Roebers, 2017; van Loon & Roebers, 2024). In reading contexts, primary school children demonstrate greater overconfidence and less accurate estimations than older students (Mirandola et al., 2018), although these processes can be improved through targeted interventions (Eberhart et al., 2025). Recent findings indicate that even within narrow age ranges, children differ substantially in their ability to evaluate their comprehension (Buehler et al., 2025; Mirandola et al., 2018). Although metacognitive abilities typically improve between the ages of 8 and 12 (Roebers, 2017), many students still demonstrate poor calibration with over or underconfident judgments, while others show better monitoring accuracy, displaying both appropriate calibration and high resolution, hence accurately distinguishing between correct and incorrect responses. However, little is known about how cognitive–linguistic factors such as vocabulary knowledge, reading fluency, and cognitive flexibility predict individual differences in monitoring calibration and resolution during middle childhood. This gap limits our understanding of the developmental mechanisms underlying metacognitive accuracy in reading comprehension and highlights the need to examine how these abilities jointly shape monitoring in naturalistic reading tasks.

### 1.3. The Present Study

This study investigates how vocabulary knowledge, reading fluency, and cognitive flexibility associates with metacognitive monitoring, specifically global calibration, item-level calibration, and resolution among fifth-grade students. Reading comprehension is examined as a potential mediator, reflecting the assumption that cognitive–linguistic resources may influence monitoring both through their impact on comprehension and in the case of fluency, through more direct heuristic pathways. To capture distinct aspects of metacognitive monitoring, the study distinguishes between global calibration (metacomprehension judgments after reading) and item-level calibration (confidence rating after each question) (Schraw, 2009; Soto et al., 2023). Global calibration reflects students’ overall confidence in their understanding immediately after reading, while item-level calibration captures their item-specific confidence judgments following each comprehension question. Global metacomprehension judgments and item-level confidence ratings capture different aspects of monitoring and can be influenced by processing fluency and other heuristic cues (Buehler et al., 2025; Koriat, 1997; Roebers, 2017; Soto et al., 2023). Including both measures allows for a comprehensive assessment of monitoring accuracy and its cognitive–linguistic associations. While calibration and resolution have been examined in both adult and child populations, research investigating how cognitive–linguistic factors relate to these monitoring abilities in naturalistic reading contexts warrants further investigation (Mirandola et al., 2018; Roebers, 2017; Soto et al., 2023). This may shed light on the developmental underpinnings of monitoring accuracy in middle childhood, a critical period marked by increasing demands for self-regulated learning and independent reading skills. To this end, the study addresses the following research question: To what extent are vocabulary knowledge, reading fluency, and cognitive flexibility associated with metacognitive monitoring (global calibration, item-level calibration, and resolution) in fifth grade reading comprehension, and are these associations mediated by comprehension performance?

While we do not assume a single explanatory pathway, prior theoretical and empirical work provides a basis for several exploratory hypotheses. First, substantial individual differences are expected in both calibration and resolution, consistent with findings that metacognitive monitoring varies widely among students even within the same age group (Mirandola et al., 2018). Second, we expect that vocabulary knowledge and reading fluency will each be positively associated with reading comprehension, given their central role in supporting meaning construction (Juhkam et al., 2023; Perfetti, 2007). Cognitive flexibility is also expected to show positive, but more modest association with comprehension. Third, reading comprehension is expected to partially or fully mediate the relationship between these cognitive–linguistic abilities and metacognitive monitoring. Specifically, vocabulary knowledge is expected to be positively associated with both global and item-level calibration and with resolution via reading comprehension, whereas reading fluency is expected to show positive indirect associations with resolution through comprehension alongside direct positive associations with calibration error, reflecting heuristic fluency-based judgments (Koriat, 1997). The role of cognitive flexibility is also examined in light of its theorized contributions to adaptive cue use and self-regulation, though its impact may be reduced when language-specific skills are taken into account (Tal & Shaul, 2024). By examining global calibration and item-level calibration alongside resolution and disentangling direct and mediated pathways, this study aims to clarify how cognitive–linguistic abilities associate with metacognitive monitoring in middle childhood. The findings are intended to inform future research and guide the design of educational interventions that foster more accurate self-monitoring and support self-regulated learning in developing readers.

## 2. Materials and Methods

### 2.1. Participants

A total of 104 fifth-grade students (M age = 10.91 years, SD = 0.36) participated in the study, including 58 boys (55.8%) and 46 girls (44.2%). Most participants attended state secular schools (84.6%), with approximately half (51.9%) from schools in the lowest socioeconomic deciles (8–10). See Table 1 for complete demographic details. All participants were native Hebrew speakers and were recruited through convenience sampling from sixty elementary schools. Schools were selected to ensure variability in reading ability and socioeconomic background.

### 2.2. Materials and Measures

#### 2.2.1. Working Memory

Working memory was assessed using the Digit Span subtest from the Wechsler Intelligence Scale for Children–Third Edition (WISC-III; Wechsler, 1991), which has been used in developmental research, including Hebrew-speaking samples (e.g., Raz & Shaul, 2025; Tal et al., 2025). Digit Span shows strong construct continuity across Wechsler versions and remains a standard indicator of verbal working memory capacity (Egeland et al., 2026). The task includes forward and backward spans. A raw composite score was used to index individual differences. Reliability was adequate (α = 0.82). Working memory was included as an exploratory control variable rather than a focal predictor.

#### 2.2.2. Cognitive Flexibility

Cognitive flexibility was assessed using the **Dimensional Change Card Sort (DCCS)** from the NIH Toolbox (Zelazo et al., 2013). Students sorted stimuli by alternating dimensions (color/shape), with accuracy and reaction time recorded (α = 0.89). Cognitive flexibility was examined as a secondary exploratory variable, consistent with theoretical framing in the introduction.

#### 2.2.3. Vocabulary Knowledge

Vocabulary was measured using the **Elul Test** (Shatil et al., 2007). The test comprised thirty-five multiple-choice items; on each item, participants selected one of four illustrations that best matched a target word. Items were ordered from easier to more difficult. Administration followed standard procedures: items were discontinued after five consecutive errors, but total test time was not constrained. The total score was the number of correct responses (0–35). (α = 0.86). The vocabulary test is appropriate for use with typical elementary school readers, as it was developed on a heterogeneous sample of Hebrew-speaking students from varied socioeconomic and literacy environments. Its use is therefore not limited to students with learning difficulties.

#### 2.2.4. Reading Fluency

Reading fluency was assessed using the **TOWRE** adapted from the Test of Word Reading Efficiency (TOWRE; Torgesen et al., 1999). The Hebrew version followed the procedure described in prior Hebrew reading studies (e.g., Kilim & Prior, 2025). The test includes real-word and pseudoword reading within 45 s time limits. Scores reflect correctly read words (α = 0.89–0.94). The TOWRE Hebrew adaptation is validated for typical readers in late primary school, making it appropriate for the current sample.

#### 2.2.5. Reading Comprehension

Reading comprehension was assessed using the **Developmental TAMAR Test** followed the procedure described in prior Hebrew reading studies (e.g., Kilim & Prior, 2025). This computerized test includes two short texts (150 words each) and five multiple-choice questions per text. Students read silently and answered questions within 10 min, with texts available during questioning. Reported reliability (α = 0.76–0.84).

#### 2.2.6. Metacognitive Monitoring in Reading Comprehension

**Metacognitive Monitoring Judgments**

Participants provided two types of metacognitive judgments during the reading comprehension task. First, global (predictive) metacomprehension judgments were collected immediately after reading each text but prior to answering comprehension questions. Participants rated their confidence in their understanding of the text on a scale from 0% (not confident at all) to 100% (completely confident). Second, item-level (postdictive) confidence ratings were obtained immediately after participants answered each comprehension question. For each response, participants indicated their confidence that their answer was correct using the same 0–100% scale, providing postdictive judgments about their performance on individual items.

**Metacognitive Monitoring Accuracy: Calibration and Resolution**

Based on the confidence judgments, metacognitive monitoring accuracy was assessed using two complementary indices: calibration and resolution. Calibration was first computed as a bias score, calculated as the difference between participants’ confidence judgments and their actual performance (confidence minus performance), indexing the direction of miscalibration. Positive values reflected overestimation (overconfidence), whereas negative values reflected underestimation (underconfidence). Calibration accuracy was then operationalized as the absolute value of this bias score, indexing the magnitude of miscalibration irrespective of direction, with values closer to zero indicating higher calibration accuracy (Schraw, 2009). Calibration accuracy served as the outcome measure in all regression and SEM analyses, whereas calibration bias was examined descriptively.

Two calibration accuracy indices were further computed to distinguish between global calibration (metacomprehension judgments after reading) and item-level calibration (confidence rating after each question): (1) **Global calibration**, derived from the predictive metacomprehension judgments averaged across both texts and calculated as the absolute difference between confidence ratings (transformed to percentages) and overall comprehension performance; and (2) **Item-level calibration**, based on postdictive confidence ratings provided after each comprehension question and calculated as the absolute difference between item-level confidence and accuracy (0 for incorrect, 100 for correct responses), then averaged across all ten questions. Calibration values closer to zero indicate better alignment between perceived and actual performance, whereas larger values indicate greater miscalibration (Dunlosky & Thiede, 2013).

**Resolution** captures participants’ ability to distinguish between correct and incorrect responses using their item-level confidence ratings provided after answering each comprehension question. It was measured using Goodman–Kruskal’s Gamma coefficient, calculated within participants across the ten comprehension questions. Given the limited number of items, Gamma was treated as an intra-individual discrimination index and interpreted with caution. Higher gamma values indicate better resolution, meaning participants are more confident in correct responses and less confident in incorrect ones. This intra-individual metric is independent of overall bias and highlights sensitivity to performance differences at the item level. This metric captures sensitivity to accuracy differences independent of absolute calibration and does not require predictive JOCs. This approach aligns with current methodological standards for assessing metacognitive monitoring accuracy in reading comprehension tasks.

### 2.3. Procedure

The study included fifth-grade students, all native Hebrew speakers, recruited from 60 elementary schools in Israel. Participants were recruited through convenience sampling in collaboration with school principals. The goal was to include classrooms representing typical heterogeneity in reading ability. Inclusion criteria required that students had no diagnosed learning disabilities, neurological disorders, or sensory impairments. All testing was conducted individually in a quiet room. Each child completed the tasks in the following fixed order: 1. Reading fluency 2. Vocabulary 3. Cognitive flexibility 4. Reading comprehension + metacognitive judgments. The order was fixed to prevent task-order effects on metacognitive judgments. Sessions lasted approximately 45–60 min. Standardized instructions were provided for all tasks, and breaks were given as needed to reduce fatigue.

### 2.4. Statistical Analysis

Sample size adequacy was evaluated using a post hoc power analysis conducted with GPower 3.1.9.7. For linear regression analyses with four predictors, assuming f^2^ = 0.15 and α = 0.05, the sample size (N = 104) yielded an achieved power of 0.888 (Cohen, 1988; Faul et al., 2009). Data analysis was conducted using SPSS 29 and AMOS 29. Preliminary analyses included the computation of descriptive statistics for all primary variables, including means, standard deviations, skewness, and kurtosis to characterize the distribution of reading comprehension, vocabulary knowledge, reading fluency, cognitive flexibility, and the metacognitive monitoring indices. Normality was examined using the Kolmogorov–Smirnov and Shapiro–Wilk tests. Multicollinearity diagnostics (Tolerance, VIF) were conducted to ensure that the predictors could be modeled simultaneously without violating independence assumptions. Intra-individual Goodman–Kruskal gamma coefficients were computed as the resolution metric, reflecting students’ ability to discriminate between correct and incorrect responses. Hierarchical regression analyses served as the primary analytic approach, considering global calibration, item-level calibration, and resolution as outcome variables. Predictors were entered in theoretically grounded blocks: vocabulary knowledge, reading fluency, and cognitive flexibility first, followed by reading comprehension to test mediation. An exploratory structural equation model (SEM) was conducted using AMOS to integrate the significant pathways identified in the regression analyses. Given the sample size, the SEM was specified as exploratory (rather than confirmatory) and is reported as supportive evidence, evaluated using standard fit indices (RMSEA, SRMR, CFI, NFI). Because all variables were measured concurrently, mediation analyses reflect statistical decomposition of associations rather than causal pathways.

**Ethical Considerations**

(The study was approved by the Chief Scientist of the Israeli Ministry of Education (12126) and the Ethics Committee for Human Research in the Faculty of Education (452/21). Written informed consent was obtained from parents, and data collection adhered to ethical guidelines ensuring anonymity.

## 3. Results

This section presents the findings from the serial regression analyses and the structural equation modeling (SEM) used to investigate how vocabulary knowledge, reading fluency, and cognitive flexibility contribute to global calibration, item-level calibration, and resolution in fifth-grade readers.

### 3.1. Individual Differences in Calibration Accuracy and Resolution

Substantial variability was observed across the three monitoring indices (Global calibration, Item-level calibration and Resolution). **Global calibration** bias scores ranged from −80 to 90 (M = 3.53, SD = 30.33) and **item-level calibration** bias scores ranged from –41.08 to 71.37 (M = 4.91, SD = 23.98). Across both indices, 58% of students demonstrated overconfidence (positive values), whereas 42% showed underconfidence. These proportions reflect descriptive directional bias (i.e., the direction of miscalibration).

**Resolution** scores ranged from −1.00 to 1.00 (M = 0.33, SD = 0.67; Md = 0.50). Accordingly, resolution values are interpreted as indicative patterns rather than precise individual estimates. Notably, 19% of students exhibited inverse resolution (negative Gamma), whereas 27% achieved perfect resolution (Gamma = 1.00), revealing pronounced heterogeneity in students’ ability to discriminate between correct and incorrect responses. These resolution findings are consistent with developmental research indicating that metacognitive monitoring abilities, including discrimination between correct and incorrect responses, continue to develop throughout elementary school years, with considerable individual differences in monitoring accuracy even within narrow age ranges (Koriat & Ackerman, 2010; Mirandola et al., 2018; Roebers, 2017; van Loon & Roebers, 2024). The wider dispersion observed for global calibration compared to item-level calibration is consistent with evidence that these judgments rely on partly distinct monitoring processes. Global metacomprehension judgments draw more on general heuristic cues, whereas item-level confidence ratings are more closely tied to local response experiences (Koriat, 1997; Roebers, 2017; Schraw, 2009). Collectively, these findings highlight marked heterogeneity in fifth graders’ metacognitive monitoring abilities across both calibration and resolution measures. Inspection of the Gamma distribution indicated broad variability without excessive clustering at boundary values and without a high proportion of tied pairs. Notably, consistent patterns were observed when examining rank-order associations between confidence and accuracy, indicating that the results were not dependent on the specific Gamma formulation. This variability provides an opportunity to examine the cognitive–linguistic factors that may account for individual differences in monitoring accuracy during this critical developmental period.

### 3.2. Predictors of Metacognitive Monitoring

To better understand the interplay among predictors of metacognitive monitoring (global calibration, item-level calibration, and resolution) and consistent with our theoretical hypotheses, we first conducted a series of regression analyses examining each of the three monitoring outcomes as well as reading comprehension (see Table 2).

Table 2 presents the standardized regression coefficients predicting global calibration, item-level calibration, resolution, and reading comprehension from the cognitive–linguistic predictors. As shown, reading comprehension accounted for substantial variance across all monitoring outcomes, while vocabulary knowledge and reading fluency showed distinct patterns of association with calibration and resolution.

Before entering predictors into the regression models, we verified that the assumption of no multicollinearity was met. Tolerance values were all above 0.20 (range: 0.674–0.970), and variance inflation factor (VIF) values were below 2 (range: 1.031–1.484), indicating no concerns regarding multicollinearity. The regression analyses accounted for a moderate proportion of variance in all outcomes. Reading comprehension emerged as the strongest predictor across monitoring indices, demonstrating large negative associations with global and item-level calibration and a positive association with resolution. Vocabulary strongly predicted reading comprehension, and reading fluency showed a weaker but still significant contribution. Working memory and Cognitive flexibility did not significantly predict any metacognitive monitoring outcome. Working memory was included in the initial regression analyses as an exploratory executive-function predictor. However, it did not show significant associations with any metacognitive monitoring outcome or with reading comprehension (see Table 2) and was therefore not retained in the final regression models or in the exploratory SEM.

These preliminary findings guided the development of the Structural Equation Model (SEM), which included only the significant associations identified in the regression analyses in order to build a theoretically justified and parsimonious model. Structural Equation Modeling (SEM) was then conducted to examine the direct and indirect relationships among cognitive–linguistic predictors and metacognitive monitoring outcomes (see Table 3).

Model fit indices for the exploratory SEM are reported in Table 3. All indices indicated numerically acceptable model fit according to conventional criteria. Given the sample size, the SEM was specified as exploratory and is interpreted as providing supportive, pattern-level evidence for the regression findings rather than as a definitive or causal test of the proposed pathways (Kumar & Upadhaya, 2017). Prior to estimation, assumptions of multivariate normality, absence of missing data, and adequate model specification were verified. The final SEM sample included 89 students with complete data. Of the original 104 participants, 15 (14.4%) had missing data due to a software malfunction during data collection. No significant differences were found between included and excluded participants on key study variables (all *p*s > 0.05). Given the modest proportion of missing data and the absence of systematic differences, listwise deletion was considered appropriate. The SEM included six observed variables, with corresponding error terms specified for each endogenous variable. The model showed numerically acceptable fit across standard indices (Mueller & Hancock, 2011), χ^2^(4) = 3.454, *p* = 0.485; CMIN/DF = 0.714; RMSEA = 0.000; SRMR = 0.032; NFI = 0.980; CFI = 1.000. Given the model’s low degrees of freedom, these global fit indices may appear near perfect and are therefore not interpreted as strong evidence. Accordingly, the SEM is best viewed as an exploratory path-analytic summary of the regression findings.

The SEM revealed clear patterns of direct, indirect, and total effects (see Table 4). Vocabulary and reading fluency both showed significant direct associations with reading comprehension. Reading fluency also showed significant direct associations with global and item-level calibration, indicating greater miscalibration and a tendency toward overconfident judgments among more fluent readers. Reading comprehension strongly predicted all dimensions of metacognitive monitoring. It negatively predicted both global and item-level calibration, suggesting that better comprehension is associated with more accurate (i.e., less inflated) self-evaluations, and positively predicted resolution, indicating that students with stronger comprehension were better able to distinguish between correct and incorrect responses.

**Figure 1:**
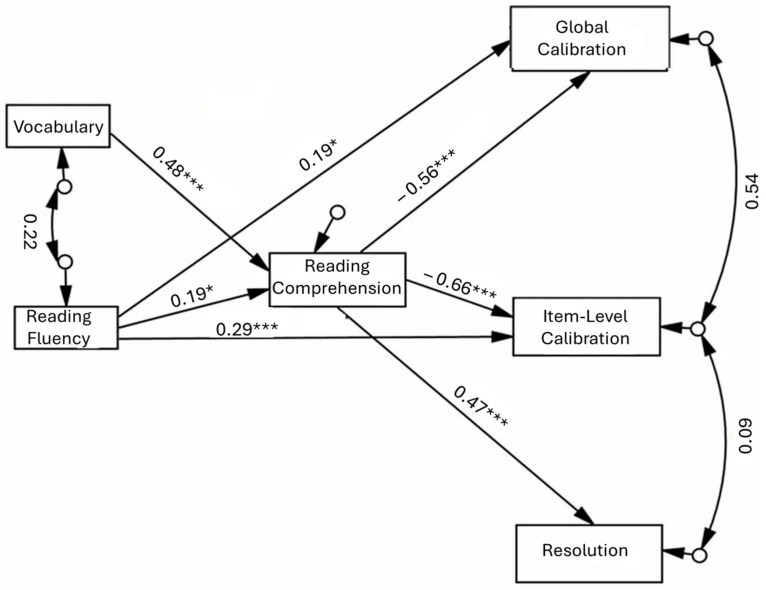
SEM of Factors Predicting Metacognitive Monitoring: Full Model. * *p* < 0.05; *** *p* < 0.001.

The model also revealed significant indirect pathways. Reading fluency was indirectly associated with global calibration, item-level calibration, and resolution through its contribution to reading comprehension. Vocabulary also showed significant indirect effects on these metacognitive monitoring outcomes, fully mediated by reading comprehension (see Figure 1). Given the cross-sectional design, these paths should be interpreted as associative rather than directional. These findings suggest that the association of vocabulary with monitoring accuracy operates primarily through the level of reading comprehension, whereas the association of reading fluency is only partially mediated for calibration and fully mediated for resolution. When total effects were considered, reading fluency showed a small but significant positive total effect on resolution, whereas its total effects on the two calibration indices did not reach significance.

Taken together, the regression and SEM analyses provide converging patterns of associations suggesting that reading comprehension is closely linked to monitoring accuracy in fifth-grade readers through cognitive–linguistic skills. Vocabulary knowledge was associated with monitoring primarily through its relation to comprehension, whereas fluency showed both direct and comprehension-related associations with calibration. These findings highlight the centrality of reading comprehension and point to potentially distinct pathways through which vocabulary and fluency are related to monitoring processes. However, given the correlational design, alternative interpretations cannot be ruled out, including shared underlying cognitive resources or reciprocal relations among reading skills and monitoring accuracy.

## 4. Discussion

The current study examined how vocabulary knowledge, reading fluency, cognitive flexibility, and working memory are associated with individual differences in metacognitive monitoring during reading comprehension among fifth-grade students. Monitoring accuracy was assessed using calibration and resolution. Calibration was measured globally through metacomprehension judgments after reading, and at the item-level through confidence ratings after each question. Resolution reflected students’ ability to discriminate between correct and incorrect responses using item-level confidence ratings. The findings reveal substantial heterogeneity in students’ ability to evaluate their comprehension performance, with patterns of both over and underconfidence, as well as a wide range of discrimination accuracy. These differences likely reflect that global metacomprehension judgments rely more on general heuristic cues, whereas item-level confidence ratings are tied more closely to local response experiences and immediate performance feedback (Koriat, 1997; Schraw, 2009). Collectively, these results provide new insights into the cognitive and linguistic processes and patterns associated with self-monitoring during reading in middle childhood, highlighting the domain-specific nature of metacognitive monitoring.

### 4.1. Cognitive–Linguistic Resources Supporting Metacognitive Monitoring in Reading

Vocabulary knowledge was most strongly associated with both calibration accuracy and resolution, and these associations were statistically accounted for by reading comprehension. This pattern aligns with claims that metacognitive monitoring is closely tied to domain-specific knowledge (Händel et al., 2020; Schraw et al., 2013) and evidence that domain-specific knowledge is often a stronger predictor than general strategies (Deekens et al., 2018). Reading comprehension tasks inherently require complex linguistic processing and are therefore often characterized by increased susceptibility to miscalibration (Dentakos et al., 2019; Kleider-Tesler et al., 2022). Although some researchers propose a domain-general view of metacognitive monitoring (Jackson & Kleitman, 2014), our findings tentatively suggest that monitoring accuracy may be shaped by specific linguistic demands. However, this should be interpreted cautiously, as monitoring was not assessed in non-verbal domains.

The fully mediated effect of vocabulary is consistent with Perfetti’s (2007) lexical quality hypothesis: higher-quality lexical representations provide more reliable semantic cues for evaluating comprehension. Children with richer vocabulary may detect inconsistencies more efficiently and anchor their confidence judgments in meaningful semantic information (Wright & Cervetti, 2017). Thus, the current findings extend prior work by suggesting that vocabulary supports metacognitive monitoring primarily through its role in comprehension. Importantly, these mediation findings reflect statistical decomposition of associations rather than causal mechanisms.

Alternative explanations should be considered. Given the cross-sectional design and shared measurement context, vocabulary knowledge and reading comprehension may reflect a shared latent verbal ability, rather than a strictly mediated relationship. The lack of significant contribution from general cognitive flexibility may reflect reading comprehension’s task-specific nature, supported by Cartwright et al. (2019), who demonstrated that reading-specific flexibility (shifting among phonological, orthographic, and semantic information) predicted reading outcomes more strongly than general executive control. A similar pattern of non-significant associations pattern was observed for working memory after accounting for core linguistic skills and reading comprehension. This aligns with evidence that beyond early reading stages, working memory’s contribution is largely indirect and attenuated when core language skills are accounted for (Kim, 2020; Morris & Lonigan, 2022; Peng et al., 2018).

The null findings for cognitive flexibility should be interpreted cautiously, as the present analyses were not designed to test its potential moderating role in cue utilization. Rather, they indicate that cognitive flexibility does not show robust direct associations with monitoring accuracy once core linguistic factors are taken into account. While our study’s findings highlight the domain-specific nature of metacognitive monitoring in reading and the attenuated role of domain-general cognitive flexibility in this context, it is crucial to acknowledge that cognitive flexibility remains a fundamental aspect of general intellectual functioning and adaptive cognition (Birney & Beckmann, 2022; Schmitz & Krämer, 2023). Its broader influence on problem-solving and adaptive behavior is well-established, suggesting that its relevance to academic success extends beyond specific task monitoring, even if its direct pathways to reading metacognition were not robust in the current study.

### 4.2. Reading Fluency and Metacognitive Monitoring

Reading fluency showed a dual pattern of associations with metacognitive monitoring. Fluency showed a significant direct association with higher calibration error, suggesting that more fluent readers tended to overestimate their comprehension. This pattern is consistent with the possibility that fluent decoding is accompanied by a subjective sense of ease. This experience may be misinterpreted as understanding and may be prone to overconfident judgments, consistent with an “illusion of understanding.” However, fluency also demonstrated indirect effects: through its contribution to reading comprehension, it was associated with better resolution and reduced calibration errors. Thus, although fluent readers may be more prone to global overconfidence, their ability to discriminate between correct and incorrect responses remains intact when their comprehension is strong.

Several accounts may explain these combined findings. One possibility, consistent with cue-utilization, is that readers sometimes rely on surface-level cues such as fluency as heuristics for comprehension, potentially leading to an “illusion of understanding.” However, the observed relationship between fluency and overconfidence does not necessarily indicate that students directly use fluency as a monitoring cue. Alternative explanations may include shared underlying factors, such as general cognitive ability or working memory, or the possibility that fluency frees cognitive resources; however, neither account was supported for global calibration in the present study. While the systematic overestimation among fluent readers is consistent with the possibility that fluency might serve as a heuristic cue, the correlational nature of our data precludes definitive causal inferences about the underlying mechanisms. A further plausible explanation involves third-variable influences, such as general cognitive efficiency. Children who read more fluently may also tend to report higher confidence, which could contribute to overconfidence independently of fluency-based cue use. Accordingly, the present findings should be interpreted as consistent with, but not uniquely supporting, a cue-utilization account.

Importantly, while global calibration was affected by fluency, the ability to discriminate between correct and incorrect responses at the item level (resolution) appeared to be preserved through the comprehension pathway. This dissociation between calibration and resolution supports their theoretical distinction (Dunlosky & Thiede, 2013) and suggests the involvement of distinct underlying monitoring processes. At the same time, prior research highlights substantial individual variability among fluent readers. As demonstrated by Kasperski and Katzir (2013), while some fluent readers exhibit high confidence, others remain cautious, suggesting that fluency does not uniformly drive overconfidence. Such within-group differences underscore the importance of examining additional moderating factors, such as attention regulation or working memory, which may influence metacognitive judgment accuracy.

The negative relationship found between reading comprehension and calibration errors should be interpreted carefully. Rather than reflecting underconfidence, lower calibration errors among stronger comprehenders indicate more accurate alignment between confidence and performance, consistent with evidence that higher performers tend to be better calibrated (Kruger & Dunning, 1999; Soto et al., 2020). Taken together, while our findings are consistent with the possibility that automated processing skills may be associated with differences in global monitoring accuracy, multiple theoretical mechanisms could account for these relationships. These include the use of fluency as a monitoring cue, the influence of shared underlying factors (such as general cognitive ability), or resource allocation effects. Further research employing experimental designs is needed to distinguish among these competing explanations.

### 4.3. Developmental Considerations and Individual Differences

Fifth grade represents a transitional phase where students are expected to engage with increasingly complex texts while developing sophisticated monitoring skills. Metacognitive abilities develop significantly between ages 8–12 (Roebers, 2017), making this an ideal age group to study the emergence and refinement of self-monitoring in academic contexts. Consistent with developmental variability research (Mirandola et al., 2018), we found substantial individual differences: 58% showing overconfidence, 42% underconfidence, and wide variation in resolution (19% inverse, 27% perfect discrimination). This uneven development is consistent with theoretical models of metacognitive-performance disconnect, particularly among less skilled readers (Buratti et al., 2013; Koriat, 1997). The observed pattern of associations suggests that comprehension quality represents a central link between linguistic knowledge and metacognitive accuracy. At the same time, fluency showed a direct association with higher calibration error, indicating that more fluent readers tend to report higher confidence in their comprehension. In some cases, this confidence may exceed actual understanding, particularly when students rely on processing ease as a cue for comprehension. Together, these findings point to the value of differentiated approaches that support fluent readers in improving calibration while drawing on their relatively intact resolution skills.

### 4.4. Educational and Practical Implications

The findings suggest several educational implications for supporting both comprehension and metacognitive monitoring. Given vocabulary’s strong association with monitoring through comprehension, instruction might focus on developing rich, interconnected semantic networks rather than breadth alone. Fluent readers may benefit from explicit instruction in metacognitive calibration. Because fluency may be associated with increased confidence, instructional support can focus on helping students distinguish between processing ease and actual comprehension. This may be achieved through confidence rating practice paired with feedback. Finally, assessment practices may benefit from examining calibration and resolution separately. Students who demonstrate good resolution, but poor calibration may require different support than those with the opposite pattern.

### 4.5. Limitations and Future Directions

Several limitations should be acknowledged. As Gamma was based on ten items, resolution findings are interpreted cautiously, and distributional inspection together with rank-order robustness checks supported the observed patterns. Post-question confidence ratings may not fully capture dynamic monitoring during reading. Future studies may benefit from incorporating online measures of monitoring, such as concurrent confidence judgments, think-aloud protocols, or process-based indices (e.g., eye-tracking or response-time patterns), to capture monitoring as it unfolds during reading. In addition, the fixed order of task administration, while intended to minimize task-order effects on metacognitive judgments, did not allow for counterbalancing and may have introduced fatigue or priming effects. The Hebrew-speaking fifth-grade sample may constrain generalizability given Hebrew’s unique orthographic properties. Vocabulary and fluency measures assessed primarily receptive skills; future work should examine different vocabulary types, cross-linguistic comparisons, and monitoring across text genres. The modest sample size, particularly with respect to the exploratory SEM, limits statistical power to detect smaller or indirect effects. Accordingly, non-significant pathways, especially those involving cognitive flexibility and working memory, should be interpreted with caution. Working memory was assessed using Digit Span from an earlier Wechsler edition, reflecting normative rather than construct-related limitations. Future studies could investigate whether vocabulary interventions and calibration training improve monitoring accuracy, incorporate real-time measures like eye-tracking, and examine developmental changes longitudinally while considering affective and cultural influences.

## 5. Conclusions

This study highlights the central association between vocabulary knowledge and metacognitive monitoring accuracy during reading comprehension. While fluent decoding and executive functions such as flexibility and working memory are undoubtedly important for reading success, it is students’ semantic proficiency that appears most critical for aligning confidence with performance. The unique contribution of this study is reflected in its combined focus on domain-specific predictors of both calibration accuracy and resolution in elementary school children, thus extending theoretical models of metacognition to a younger, school-based population. The complex patterns we identified regarding fluency reveal that automated processing skills, while beneficial for comprehension, may be associated with less accurate global monitoring. Such indications may also highlight the need for differentiated instructional approaches. By highlighting vocabulary’s role and the domain-specific nature of metacognitive monitoring, these findings may inform instructional practices aimed at fostering self-regulated reading. Understanding how cognitive–linguistic resources support monitoring may guide interventions that strengthen both comprehension and metacognitive accuracy.

## Figures and Tables

**Table 1:** Participants Description (N = 104).

		n	%
Gender	Boys	58	55.8
	Girls	46	44.2
School Type	State Secular	88	84.6
	State Religious	15	14.4
Socioeconomic School Decile	Decile 1–3 (Strongest)	10	9.6
	Decile 4–7 (Average)	39	37.5
	Decile 8–10 (Weakest)	54	51.9
Age	M (SD)	10.91 (0.36)	

**Table 2:** Standardized Regression Coefficients Predicting Metacognitive Monitoring and Reading Comprehension.

Variable	Global Calibration R^2^ = 0.393	Item-Level Calibration R^2^ = 0.513	Resolution R^2^ = 0.255	Reading Comprehension R^2^ = 0.323
Reading Comprehension	−0.715 **	−0.767 **	0.396 **	-
Working Memory	0.028	0.066	0.095	−0.003
Vocabulary Knowledge	0.113	0.007	0.148	0.473 **
Reading Fluency	0.176 *	0.251 *	−0.070	0.221 *

* *p* < 0.05; ** *p* < 0.01.

**Table 3:** Goodness-of-fit indices for the SEM of factors associated with metacognitive monitoring of 5-th grade students.

	CMIN/DF	RMSEA	SRMR	NFI	CFI
Recommended Values	<2	<0.06	<0.08	>0.90	>0.95
Model	0.714	0.000	0.032	0.980	1.000

RMSEA = root mean square error of approximation; SRMR = standardized root mean square residual; NFI = normed fit index; CFI = comparative fit index.

**Table 4:** Direct, Indirect and Total Effects of Factors Predicting Metacognitive Monitoring: Corrected Model.

			β	*p*
**Direct Effects**				
Reading fluency	--->	Reading comprehension	0.190	0.037
Vocabulary	--->	Reading comprehension	0.481	<0.001
Reading fluency	--->	Global Calibration	0.192	0.041
Reading fluency	--->	Item-Level Calibration	0.290	<0.001
Reading comprehension	--->	Global Calibration	−0.561	<0.001
Reading comprehension	--->	Item-Level Calibration	−0.660	<0.001
Reading comprehension	--->	Resolution	0.474	<0.001
**Indirect Effects**				
Reading fluency--->Reading comprehension--->Resolution			0.09	0.026
Reading fluency--->Reading comprehension--->Global Calibration			−0.101	0.032
Reading fluency--->Reading comprehension--->Item-Level Calibration			−0.125	0.034
Vocabulary--->Reading comprehension--->Resolution			0.228	<0.001
Vocabulary--->Reading comprehension--->Global Calibration			−0.270	0.002
Vocabulary--->Reading comprehension--->Item-Level Calibration			−0.317	0.002
**Total Effects**				
Reading fluency	--->	Resolution	0.09	0.026
Reading fluency	--->	Global Calibration	0.085	0.398

## Data Availability

The data presented in this study are available on request from the corresponding author due to ethical and privacy restrictions.
